# Effect of smoking and smoking cessation on hematological parameters in polycythemic patients

**DOI:** 10.1515/med-2025-1181

**Published:** 2025-04-29

**Authors:** Kadir Ilkkilic, Dilek Karadogan, Tahsin Gökhan Telatar, Osman Cure, Aleksandra Klisic

**Affiliations:** Department of Hematology, School of Medicine, Recep Tayyip Erdogan University, Rize, Turkey; Department of Chest Diseases, School of Medicine, Recep Tayyip Erdogan University, Rize, Turkey; Department of Public Health, School of Medicine, Recep Tayyip Erdogan University, Rize, Turkey; Department of Rheumatology, School of Medicine, Recep Tayyip Erdogan University, Rize, Turkey; Faculty of Medicine, Montenegro University, Podgorica, Montenegro

**Keywords:** polycythemia, smoking cessation intervention, hematological parameters

## Abstract

**Background:**

The number of studies on the effect of smoking cessation interventions in polycythemic patients is limited. Our aim was to examine the effects of quitting smoking on hematological parameters in patients with polycythemia and to evaluate the outcomes of smoking cessation interventions in polycythemic patients.

**Methods:**

The prospective study was conducted in the hematology outpatient clinic between November 2022 and May 2023. Patients with polycythemia were grouped according to their smoking status. Smoking cessation interventions were applied to current smokers. Blood parameters were compared according to smoking status at the baseline and third month follow up.

**Results:**

At first presentation, 65.4% (*N* = 93) of 142 patients were current smokers and 34.5% (*N* = 49) were non-smokers. Fifty-one percent (*N* = 47) of active smokers quit smoking at the 3-month follow-up after the intervention. Hemoglobin (Hb), hematocrit (Hct), mean corpuscular volume, very low density lipoprotein cholesterol, and immature granulocytes were found to be higher in active smokers compared to non-smokers at initial presentation (*p* < 0.05). Hb and Hct values decreased in smokers who quit smoking compared to baseline values at 3 months (*p* < 0.05).

**Conclusions:**

Of the patients with polycythemia, 65% were current smokers and about half of them were successful in quitting smoking at 3 months with effective implementation of smoking cessation interventions.

## 1 Introduction

Smokers are exposed to carbon monoxide, nitrosamine compounds, and many types of hydrocarbons, resulting in a significantly increased risk of cancer, cardiovascular diseases, and respiratory diseases. Polycythemia is a condition characterized by an increase in red blood cell (RBC) volume and hemoglobin (Hb) level. The condition presents as primary polycythemia as a result of hereditary or acquired mutation or as secondary polycythemia characterized by an increase in erythropoietin levels and increased RBC production due to impaired tissue oxygenation. Carbon monoxide taken in with cigarette smoke binds to Hb and forms carboxyhemoglobin, which has no oxygen carrying capacity, and causes hypoxia by decreasing the oxygen carrying capacity to tissues. To prevent tissue hypoxia, erythropoietin secretion increases and erythrocyte production is induced in the bone marrow as a compensatory mechanism [1]. Toxic substances in tobacco have been found to be associated with clonal hematopoiesis by strongly stimulating inflammation and increasing oxidative stress [2]. It has been shown that cigarette smoke is a potent inflammatory stimulus via nuclear factor-κB and Jakus kinase (JAK) pathways and contributes to leukocytosis by strongly increasing neutrophil and interleukin 8 (IL-8) production [3]. In addition, smoking has been found to increase inflammatory cytokines such as IL-6 and tumor necrosis factor alpha [4]. It is thought to cause deterioration in hematological parameters through these pathways. Cases of secondary polycythemia as a result of systemic hypoxia due to smoking are also very common, and studies have shown a strong relationship between smoking and secondary polycythemia [5,6]. In addition to polycythemia, there are also studies showing that smoking increases hematological inflammatory parameters such as neutrophils and lymphocytes and causes triggering of inflammation, endothelial dysfunction, atherosclerosis, and chronic respiratory diseases [2,6,7,8].

Although efforts to prevent tobacco use in most developed countries are effective, the problem of tobacco use continues worldwide [9]. Smoking cessation interventions for the prevention of tobacco use have been shown to increase smoking cessation rates [10]. In addition, it was found that advice to quit for medical reasons increased the frequency of smoking cessation attempts [11]. In the literature, there are studies showing the link between polycythemia and tobacco exposure, but the effect of smoking cessation interventions in polycythemic patients and its effects on blood parameters are not well addressed in previous studies.

Therefore, we aimed to examine the effects of smoking cessation on hematological parameters in polycythemic patients and to evaluate the outcomes of smoking cessation interventions in polycythemic patients.

## 2 Materials and methods

Our study was conducted on patients who visited the hematology outpatient clinic at the Recep Tayyip Erdoğan University Training and Research Hospital between November 2022 and May 2023.

In this prospective cohort study, patients aged 18–80 years with polycythemia were included. Polycythemia is defined as the Hb level above 16.5 g/dL in men and 16 g/dL in women or hematocrit (Hct) >49% in men and >48% in women according to the World Health Organization criteria [12].

Informed consent was obtained from the patients participating in the study. A follow-up form was filled out regarding the smoking and quitting status of the patients at baseline and during follow-up.

Questions were asked regarding demographic data (age, gender, employment status, educational status, income level, etc.), presenting complaints and comorbidities. In addition, smoking behavior and the relationship between polycythemia and tobacco exposure and their level of knowledge were recorded by face-to-face questioning. Previous phlebotomy and blood donation status were questioned.

After fasting for at least 8–12 h, complete blood count (including immature granulocytes), lipid profile, glucose, uric acid, albumin, total protein, iron, total iron binding capacity (TIBC), ferritin, folate, total bilirubin, direct bilirubin, lactate dehydrogenase (LDH), aspartate aminotransferase (AST), alanine aminotransferase (ALT), calcium, and gamma-glutamyl transferase (GGT) were analyzed.

In the next step of study, smoking cessation interventions were administered to all patients who smoked, regardless of whether they volunteered to quit smoking [13]. Immediate appointments were scheduled for patients at the smoking cessation outpatient clinic [14]. Details of practices in the smoking cessation outpatient clinic are available in our previous article [15]. Smoking cessation status of the patients was questioned at the third month follow-up and those who quit were validated by exhaled carbon monoxide measurement. At the third month follow-up of patients, the parameters studied at baseline were measured again.

At the end of the third month, those in the smoker group were divided into ex-smokers and current smokers, while nonsmokers remained as nonsmokers.

### 2.1 Exclusion criteria

Patients with a diagnosis of cancer, hematological diseases other than secondary polycythemia, active psychiatric disease, obstructive/restrictive lung disease or obstructive sleep apnea syndrome, living at a high altitude, need for phlebotomy during the 3-month follow-up, cyanotic congenital heart disease, liver or kidney disease, androgen therapy, and alcohol use were excluded from the study. Patients who were found to be JAK2-positive and fulfilled the criteria for the diagnosis of polycythemia vera were excluded from the study.

### 2.2 Statistical analysis

The data of the study were analyzed with SPSS 29.0 software. Continuous data were presented with mean and standard deviation (SD) values, and categorical data were presented with number and percentage values. The relationships between smoking status and some socio-demographic characteristics of the participants were evaluated with the independent samples *t* test and chi-square tests. A comparison of first application baseline laboratory values of smoker and non-smoker groups was performed with the independent samples *t* test. The ANOVA test was used to evaluate the relationship between laboratory parameters and smoking cessation status of the groups at 3 months. A comparison of laboratory parameters of three groups separately according to pre- and postintervention periods was analyzed by the paired samples *t* test. There are no missing data in the variables included in the analysis. Statistical significance level *p* < 0.05 was accepted in all analyses.


**Informed consent statement:** Informed consent has been obtained from all individuals included in this study.
**Ethical approval:** Ethics committee approval was obtained for this study from the Faculty of Medicine at Recep Tayyip Erdogan University, non-interventional clinical research ethics committee chairmanship (approval number: 2022/176; approval date: 13/10/2022). The study was conducted according to the Declaration of Helsinki.

## 3 Results

A total of 142 patients with polycythemia were examined. Of these, 138 were male and 4 were female. At initial presentation, 93 patients were smokers and 49 were non-smokers. At the end of the third month, those in the smokers group were divided into ex-smokers (*N* = 47) and current smokers (*N* = 46), while the non-smokers remained as non-smokers (*N* = 49).

The mean age was 46.2 ± 14.5 years (minimum 20 to maximum 79). The most common reasons for presentation were headache, fatigue, and burning in the extremities. Hypertension and diabetes mellitus were the most common comorbidities. When baseline characteristics were compared according to smoking status, headache and tinnitus were more common in smokers and fatigue was more common in non-smokers (*p* < 0.05) (Table 1).

**Table 1:** Demographic characteristics of patients

	Total (*N* = 142)	Smoker (*N* = 93)	Non-smoker (*N* = 49)	*p*-value
Age, years	46.27 ± 14.54	46.53 ± 13.74	45.78 ± 16.10	0.771
Age group				0.593
≤44	62 (43.7%)	39 (62.9%)	23 (37.1%)	
45–64	63 (44.3%)	44 (69.8%)	19 (30.2%)	
≥65	17 (12.0%)	10 (58.8%)	7 (41.2%)	
Gender				0.141
Female	4 (3%)	4 (4.3%)	0	
Male	138 (97%)	89 (95.7%)	49 (100%)	
Employment status				0.633
Unemployed	56 (39%)	38 (67.9%)	18 (32.1%)	
Employed	86 (61%)	55 (64.0%)	31 (36.0%)	
Education Status				0.093
Primary education	52 (36.6%)	40 (76.9%)	12 (23.1%)	
High school	56 (39.4%)	33 (58.9%)	23 (41.1%)	
University	34 (24%)	20 (58.8%)	14 (41.2%)	
Income level				0.230
≤Minimum wage	57 (40.1%)	34 (59.6%)	23 (40.4%)	
>Minimum wage	85 (59.9%)	59 (69.4%)	26 (30.6%)	
Admission reasons				
Headache	55 (38.7%)	45 (81.8%)	10 (18.2%)	0.001
Dizziness	16 (11.2%)	11 (68.8%)	5 (31.3%)	0.771
Weakness	31 (21.8%)	9 (29.0%)	22 (71.0%)	<0.001
Itching	14 (9.8%)	7 (50%)	7 (50%)	0.199
High blood pressure	16 (11.2%)	5 (31.3%)	11 (68.8%)	0.002
Tinnitus	14 (9.8%)	14 (100%)	0	0.004
Hand and feet burning	27 (19%)	21 (77.8%)	6 (22.2%)	0.136
Comorbidities				
Diabetes mellitus	33 (23.2%)	21 (63.6%)	12 (36.4%)	0.798
Hypertension	50 (35.2%)	29 (58.0%)	21 (42.0%)	0.166
Hyperlipidemia	17 (12%)	10 (58.8%)	7 (41.2%)	0.538
Coronary artery disease	12 (8.5%)	7 (58.3%)	5 (41.7%)	0.586

Table 2 shows the smoking behavior of smokers and their level of knowledge about smoking. A total of 76.3% of the current smokers answered “no” to the question, “*Does smoking cause polycythemia?”*, 77.4% had attempted to quit smoking before, and 81.7% of them did not know that there was a smoking cessation outpatient clinic in their neighborhood.

**Table 2:** Level of knowledge and attitude toward smoking of smokers

Smoker (*N* = 93)				
Does smoking cause polycythemia?			Number of attempts to quit smoking, mean (SD)	0.01 (0.10)
Yes	4 (4.3%)		Is there a smoking cessation clinic in your province?	
No	71 (76.3%)		Yes	11 (11.8%)
No idea	18 (19.4%)		No	76 (81.7%)
Smoking duration (year), mean (SD)	25.6 (13.8)		No idea	6 (6.5%)
Number of cigarettes smoked per day, mean (SD)	18.9 (7.09)		Number of evidence-based cessation aids, mean (SD)	0.01 (0.10)
Package year, mean (SD)	25.6 (17.5)		Previous phlebotomy status	
Age of smoking initiation, mean (SD)	20.8 (6.02)		0	53 (57%)
Number of smokers in household, mean (SD)	0.50 (0.60)		1	26 (28%)
Does your partner smoke?			≥2	14 (15%)
Yes	40 (43.0%)		Previous blood donation status	
No	53 (57.0%)		Yes	37 (39.8%)
Fagerstrom test for nicotine dependence score, mean (SD)	3.6 (2.10)		No	56 (60.2%)
Attempts to quit smoking				
Yes	72 (77.4%)			
No	21 (22.6%)			

A comparison of baseline laboratory parameters of the patients according to smoking status is shown in Table 3. Hb, Hct, mean corpuscular volume (MCV), very low density lipoprotein cholesterol (VLDL-cholesterol), and immature granulocyte (IMG) levels were significantly higher in current smokers compared to non-smokers (*p* < 0.05). The albumin level was lower in smokers compared to non-smokers (*p* < 0.05). Total bilirubin and LDH levels were lower in smokers (*p* values <0.001 and 0.035, respectively).

**Table 3:** Comparison of first application basal laboratory values of smoker and non-smoker groups

	Current smoker (*N* = 93)	Non-smoker (*N* = 49)	*p* value
	Mean (SD)	Mean (SD)	
WBC (×10^3^/μL)	8.35 (2.16)	7.89 (1.86)	0.211
Neutrophils (×10^3^/μL)	5.05 (1.76)	4.73 (1.73)	0.300
Lymphocytes (×10^3^/μL)	2.61 (0.81)	2.38 (0.86)	0.119
Hb (g/dL)	17.4 (0.6)	17 (0.4)	0.002
Hct (%)	51.1 (2.6)	49.9 (1.7)	0.004
MCV (fL)	89.4 (4.9)	87.7 (3.6)	0.040
Platelets (×10^3^/μL)	236 (61)	242 (53)	0.602
IMG count	0.02 (0.02)	0.01 (0.01)	0.010
IMG (%)	0.31 (0.26)	0.21 (0.17)	0.022
Total bilirubin (mg/dL)	0.72 (0.29)	1.00 (0.47)	<0.001
Direct bilirubin (mg/dL)	0.13 (0.13)	0.14 (0.06)	0.794
LDH (U/L)	183.85 (43.29)	200.28 (42.30)	0.035
Albumin (g/dL)	4.48 (0.27)	4.58 (0.27)	0.043
Total cholesterol (mg/dL)	206.05 (44.6)	197.94 (40.73)	0.361
Triglycerides (mg/dL)	199.41 (132.02)	178.66 (131.52)	0.440
HDL-cholesterol (mg/dL)	41.94 (9.38)	45.51 (8.96)	0.063
LDL-cholesterol (mg/dL)	129.55 (38.72)	118.48 (31.93)	0.145
VLDL-cholesterol (mg/dL)	43.02 (29.89)	28.16 (11.56)	0.045

Abbreviations: WBC: white blood cell; MCV: mean corpuscular volume; IMG: immature granulocyte; LDH: lactate dehydrogenase; HDL: high density lipoprotein; LDL: low density lipoprotein; VLDL: very low density lipoprotein.

Table 4 shows the laboratory parameters with significant difference according to the smoking cessation status of the groups at the third month. White blood cell (WBC) and neutrophil levels were found to be higher in current smokers than in never smokers (*p* value, 0.036 and 0.031, respectively).

**Table 4:** Laboratory parameters with significant differences according to the smoking cessation status of the groups at 3 months

	Ex-smoker (*N* = 47)	Current smoker (*N* = 46)	Non-smoker (*N* = 49)	*p* value
	Mean (SD)	Mean (SD)	Mean (SD)	
WBC (×10^3^/μL)	8.16 (1.79)	8.80 (2.48)*	7.75 (1.52)*	0.036
Neutrophils (×10^3^/μL)	4.83 (1.65)	5.38 (2.04)*	4.49 (1.15)*	0.031
Monocytes (×10^3^/μL)	0.60 (0.16)*	0.57 (0.16)	0.52 (0.13)*	0.029
RDW (%)	13.26 (0.71)*	13.74 (0.78)*^#^	13.31 (0.69)^#^	0.003
Calcium (mg/dL)	9.68 (0.34)*^#^	9.93 (0.46)*	9.98 (0.036)^#^	0.004
ALT (U/L)	27 (11)*	30 (16)	36 (23)*	0.041
GGT (U/L)	29 (15)*	99 (81)*^#^	40 (15)^#^	0.003

*, ^#^ indicate groups with significant differences between them. Abbreviations: WBC: white blood cells; RDW: red cell distribution width; ALT: alanine aminotransferase; GGT: gamma-glutamyl transferase.

In Table 5, the laboratory parameters of the three groups were compared separately according to the periods before and after the intervention. In the smoking cessation group, Hb and Hct values decreased in the third month compared to baseline values (*p* < 0.001). In current smokers, the LDH level increased significantly in the third month compared to baseline values (*p* < 0.05).

**Table 5:** Comparison of laboratory parameters of three groups separately according to pre- and postintervention periods

Participants	Ex-smoker (*N* = 47)			Current smoker (*N* = 46)			Non-smoker (*N* = 49)		
Variable	First application	Third month control	*p* value	First application	Third month control	*p* value	First application	Third month control	*p* value
	Mean (SD)	Mean (SD)		Mean (SD)	Mean (SD)		Mean (SD)	Mean (SD)	
WBC (×10^3^/μL)	8.10 (1.78)	8.16 (1.79)	0.824	8.61 (2.49)	8.80 (2.48)	0.672	7.89 (1.86)	7.75 (1.52)	0.581
Neutrophils (×10^3^/μL)	4.75 (1.59)	4.83 (1.65)	0.799	5.36 (1.88)	5.38 (2.04)	0.936	4.73 (1.73)	4.49 (1.15)	0.317
Lymphocytes (×10^3^/μL)	2.52 (0.77)	2.48 (0.66)	0.655	2.69 (0.84)	2.55 (0.90)	0.092	2.38 (0.86)	2.40 (0.73)	0.795
Hb (g/dL)	17.6 (0.7)	17 (0.9)	<0.001	17.1 (0.5)	17.2 (0.7)	0.882	17 (0.4)	17.2 (0.6)	0.08
Hct (%)	51.9 (2.6)	50 (3)	<0.001	50.2 (2.3)	50 (2.2)	0.490	49.9 (1.7)	50.2 (2)	0.220
MCV (fL)	90 (4.9)	89.4 (5.2)	0.130	88.8 (4.9)	89.2 (5)	0.144	87.7 (3.6)	87.5 (3.3)	0.406
Platelets (×10^3^/μL)	230 (58)	238 (60)	0.064	243 (63)	240 (60)	0.459	242 (53)	245 (53)	0.531
AST (U/L)	24 (12)	21 (7)	0.180	23 (8)	25 (10)	0.319	27 (11)	26 (14)	0.512
ALT (U/L)	32 (15)	27 (11)	0.038	32 (16)	30 (16)	0.139	38 (24)	36 (23)	0.434
LDH (U/L)	185 (44)	183 (45)	0.771	180 (40)	200 (47)	0.030	199 (42)	192 (36)	0.298
Iron (µg/dL)	130 (60)	101 (42)	0.302	100 (41)	102 (52)	0.877	118 (46)	120 (47)	0.826
TIBC (µg/dL)	346 (52)	343 (37)	0.844	332 (62)	341 (62)	0.319	341 (43)	342 (51)	0.900
Ferritin (ng/mL)	126 (57)	95 (32)	0.067	104 (67)	88 (48)	0.065	83 (66)	84 (60)	0.908

Abbreviations: WBC: white blood cell; MCV: mean corpuscular volume; AST: aspartate aminotransferase; ALT: alanine aminotransferase; LDH: lactate dehydrogenase.

## 4 Discussion

In our study, 65% of the polycythemia patients were current smokers. Most of them were willing to quit smoking and 51% of them were able to quit smoking at the end of the third month with cessation interventions and immediate appointment arrangement to the smoking cessation clinic. Mean Hb and Hct values were found to be higher at baseline in smokers than in non-smokers and decreased significantly in the group that quit smoking at the third month follow-up. However, no difference was observed in the group that continued to smoke.

In our study, the fact that Hb levels were found to be higher in smokers at first presentation indicates a positive relationship between smoking and severity of polycythemia.

Free radicals, peroxides, and carbon monoxide in cigarette smoke are clearly linked to pathophysiological events such as prostaglandin and thromboxane synthesis and affect hemostasis. It is also known to play a role in the pathogenesis of many diseases, including atherosclerosis, carcinoma, and inflammatory diseases [16,17].

While the majority of polycythemia patients continued to smoke, 51% of the patients quit smoking with our interventions. Smoking cessation rates increase with the effective evaluation of these processes, which are “teachable moments for patients,” by the primary responsible physician [18].

In a study in which behavioral therapy and nicotine replacement therapy (NRT) were recommended in addition to medical reasons in smoking cessation interventions, it was shown that the effectiveness was higher [11]. In our study, NRT was started in addition to behavioral therapy in patients who attended the smoking cessation outpatient clinic, and 51% of the patients were able to quit smoking. This showed that the combination of NRT and behavioral therapy is an effective method for smoking cessation. In addition, 81.7% of the smokers in our study were unaware of the existence of a smoking cessation outpatient clinic in their region. Therefore, there is a need to inform patients about the existence of smoking cessation outpatient clinics in order to increase the application rate of polycythemia patients to smoking cessation outpatient clinics and the chance of success in smoking cessation.

In a study conducted by Malenica et al., the effect of smoking on hematological parameters in a healthy population was examined, and it was stated that it caused negative effects on Hb, WBC, and mean corpuscular Hb and this may cause serious consequences such as cardiovascular diseases, chronic obstructive pulmonary disease (COPD), and atherosclerosis [17]. In addition, in another study in which lipid profile disorders were shown in smokers, it was reported that these changes may lead to fatal heart diseases [19]. In our study, Hb and WBC values as well as VLDL-cholesterol levels were found to be higher in smokers at initial presentation, but there was no significant change in triglyceride, LDL-cholesterol, and HDL-cholesterol levels.

In smokers, carbon monoxide exposure results in increased production of carboxy Hb and tissue hypoxia. This results in an increase in erythropoietin secretion and thus an increase in erythrocyte count and Hct values [1]. One study found that higher RBC and Hct levels in smokers were associated with increased blood viscosity and coagulation. It was also observed that increased Hct was associated with progression of atherosclerosis and increased risk of cardiovascular diseases. In the same study, it was observed that Hct levels returned to normal within 5 years after patients quit smoking [20]. In our study, it was found that the Hb level was higher in the smoking group, and there was a significant decrease in Hb and Hct levels after smoking cessation in the third month control. This showed us that smoking cessation may improve treatment success in polycythemic patients. The significant decrease in Hb and Hct values after smoking cessation in patients will reduce the need for additional therapeutic interventions such as phlebotomy and reduce serious complications such as thrombosis. In addition, it will reduce the symptoms associated with polycythemia such as headache, fatigue, and tinnitus. In the long term, smoking cessation in patients with polycythemia will contribute to a reduction in smoking-related cardiovascular diseases, atherosclerosis, COPD, malignancies, and inflammatory diseases.

The increase in leukocyte count in smokers may be explained by the systemic inflammatory response caused by activation of proinflammatory cytokines. In addition, the increase in MCV value may occur as a result of smoking-related oxidative stress or free radical formation causing changes in the erythrocyte surface [21]. In one study, it was observed that leucocyte values decreased over time with smoking cessation [22]. In our study, WBC and neutrophil values were found to be higher in patients who continued to smoke compared to non-smokers at the third month control, but no significant decrease was observed in those who quit smoking. This may be related to the short follow-up period. The MCV value was observed to be higher in smokers at the first presentation and was consistent with the literature.

IMG are premature granulocytes released from the bone marrow and have been shown to increase in cases of inflammation and infection [23,24]. In our study, we observed that the number and percentage of IMGs were higher in smokers, and this may be an indicator of smoking-related inflammation. Albumin levels were lower in smokers, and it has been suggested that low albumin levels may be a marker of susceptibility to smoking-induced inflammatory response [25]. In our study, the albumin level was found to be low in polycythemia patients who smoked at initial presentation, and this may again be related to chronic inflammation.

### 4.1 Strength and limitations

Our study is important in terms of the methods to be used to reduce the adverse effects of tobacco use on Hb and Hct in polycythemia patients. To our knowledge, this is the first study to demonstrate the effectiveness of smoking cessation interventions in polycythemia patients. The limitations of our study are that the number of patients was limited, female smokers were very few, and the study was conducted in a single center.

### 4.2 Suggestions for further research

In our study, we investigated the effects of smoking and smoking cessation on hematological parameters in polycythemia patients and emphasized the importance of smoking cessation and intervention. Future studies may add a different dimension to this issue by integrating artificial intelligence (AI) applications. In recent years, AI has been increasingly used in medicine, especially in molecular biology, drug discovery, and clinical diagnosis [26]. Breakthroughs in protein structure prediction with the AlphaFold model provide important tools for drug design and disease mechanism research, while clinical image-based AI models show great potential in individualized diagnosis and treatment [27]. The application of these technologies provides new perspectives and methods for medical research and can significantly improve the efficiency and accuracy of research. In this study, we investigated the effect of smoking on hematological parameters in patients with polycythemia, and AI technology can determine the complex relationship between smoking and hematological parameters by analyzing large amounts of clinical data. For example, AI models can be used to predict the effects of smoking on different hematological parameters and may even help design personalized smoking cessation intervention programs. As noted in our study, 51% of the smokers successfully quit smoking after receiving smoking cessation interventions. AI technology can provide personalized smoking cessation advice and intervention strategies by analyzing patients’ smoking behavior, physiological parameters, and psychological state.

## 5 Conclusions

The present findings show a significant decrease in Hb level in polycythemia patients with smoking cessation. Developing and improving the effectiveness of smoking cessation interventions is crucial to help reduce the burden of smoking-related polycythemia and improve the outcomes. One of the possible reasons for the high success rate of smoking cessation in our study is that the patients were informed by the physician that tobacco exposure is one of the important causes of polycythemia. Therefore, polycythemic smokers should be actively encouraged to quit smoking, their awareness should be raised, and effectiveness should be monitored after the intervention. In this way, it should be aimed to better treat polycythemia in patients and to reduce the symptoms and life-threatening complications associated with polycythemia.
